# Unexpected extraluminal omental bleeding during endoscopic full-thickness resection for a gastric subepithelial lesion

**DOI:** 10.1055/a-2362-1106

**Published:** 2024-07-29

**Authors:** Chaoqin Wang, Suhuan Liao, Silin Huang, Bo Li, Guang Yang, Jianzhen Ren, RenJie Chang

**Affiliations:** 1 Spleen and Stomach Diseases, Yunnan Provincial Hospital of Traditional Chinese Medicine, Medical School, Yunnan University of Chinese Medicine, Yunnan, China; 2 Department of Gastroenterology, South China Hospital, Medical School, Shenzhen University, Shenzhen, China

Endoscopic full-thickness resection (EFTR) has become the treatment of choice for subepithelial lesions (SELs) that originate from the muscularis propria and/or exhibit exophytic growth patterns 1 . Bleeding is a recognized risk associated with endoscopic resection procedures, occurring both intraoperatively and postoperatively, predominantly from the resection site 2 , with electrocoagulation a well-established technique for achieving hemostasis 3 4 . There have been no previous reports of omental bleeding caused by EFTR. We report a case of extraluminal omental bleeding induced during EFTR for a gastric SEL, which was successfully managed with endoscopic hemostasis ( Video 1 ).

Unexpected omental bleeding occurring during endoscopic full-thickness resection for a subepithelial lesion is successfully managed with endoscopic electrocoagulation.Video 1

A 41-year-old woman underwent gastroscopy, which revealed an 8-mm SEL in the upper gastric body ( Fig. 1 ). She was hospitalized and subsequently underwent EFTR. Intraoperatively, it was confirmed that the lesion was originating from the muscularis propria, with significant exophytic growth ( Fig. 2 **a** ). Unexpectedly, during the resection, there was a sudden influx of blood into the stomach from the abdominal cavity, with no bleeding observed at the incision site. Once the expeditious and complete removal of the lesion had been completed ( Fig. 2 **b** ), active bleeding from the omentum was identified, located extraluminally to the stomach wall ( Fig. 3 **a** ). Consequently, we used disposable hemostatic forceps for electrocoagulation (Soft coagulation, effect level 4, power 80 W) ( Fig. 3 **b** ) to achieve successful hemostasis ( Fig. 3 **c** ), ultimately closing the incision with a nylon suture and clips ( Fig. 3 **d** ). Postoperatively, the patient received antibiotic therapy for 72 hours, and serial hematologic assessments showed no decline in her hemoglobin levels. The patient was discharged 3 days postoperatively, without any other complications having occurred.

**Fig. 1:**
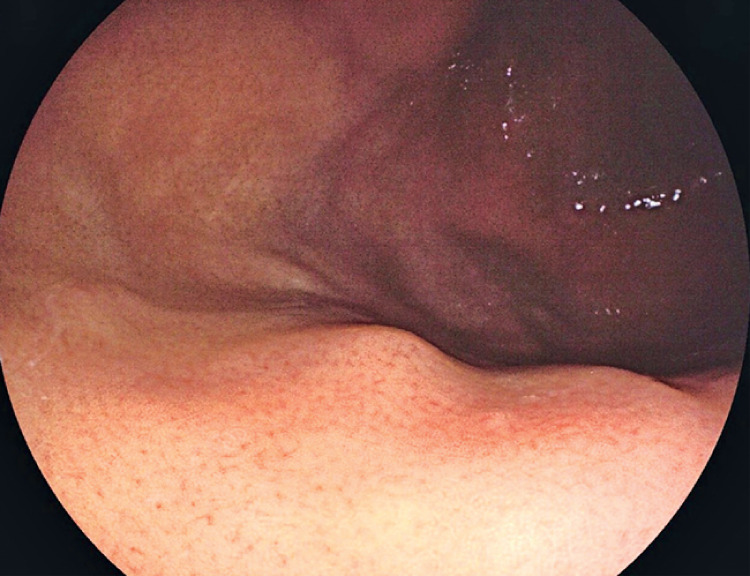
Endoscopic view showing a subepithelial lesion, measuring approximately 8 mm in diameter, in the upper part of the gastric body.

**Fig. 2:**
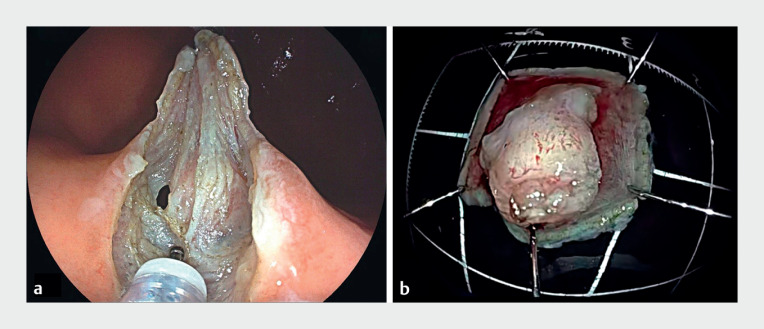
Appearance of the tumor: **a** during the endoscopic full-thickness resection procedure; **b** following resection.

**Fig. 3:**
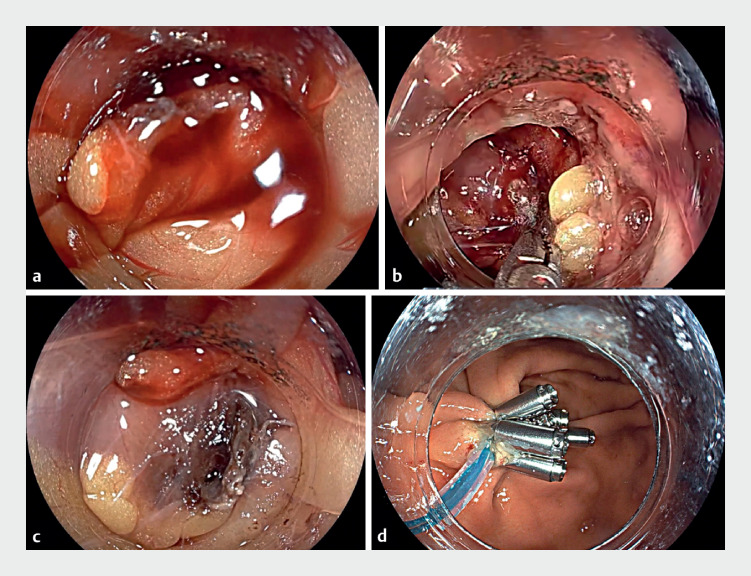
Endoscopic views showing: **a** continuous extraluminal omental bleeding; **b** endoscopic electrocoagulation being performed; **c** the appearance after successful hemostasis; **d** closure of the defect with a nylon suture and metal clips.

To the best of our knowledge, this is the first report of omental bleeding induced by EFTR and successfully managed with endoscopic electrocoagulation; it provides valuable insights into the management of complications arising from endoscopic therapeutic interventions.

Endoscopy_UCTN_Code_CPL_1AH_2AZ_3AF

## Notes

Endoscopy E-Videos https://eref.thieme.de/e-videos
*E-Videos* is an open access online section of the journal *Endoscopy* , reporting on interesting cases and new techniques in gastroenterological endoscopy. All papers include a high-quality video and are published with a Creative Commons CC-BY license. Endoscopy E-Videos qualify for HINARI discounts and waivers and eligibility is automatically checked during the submission process. We grant 100% waivers to articles whose corresponding authors are based in Group A countries and 50% waivers to those who are based in Group B countries as classified by Research4Life (see: https://www.research4life.org/access/eligibility/ ). This section has its own submission website at https://mc.manuscriptcentral.com/e-videos .
